# Diversity of Bacteria Carried by Pinewood Nematode in USA and Phylogenetic Comparison with Isolates from Other Countries

**DOI:** 10.1371/journal.pone.0105190

**Published:** 2014-08-15

**Authors:** Diogo Neves Proença, Luís Fonseca, Thomas O. Powers, Isabel M. O. Abrantes, Paula V. Morais

**Affiliations:** 1 IMAR-CMA, University of Coimbra, Coimbra, Portugal; 2 Plant Pathology Department, University of Nebraska–Lincoln, Lincoln, Nebraska, United States of America; 3 Department of Life Sciences, FCTUC, University of Coimbra, Coimbra, Portugal; CEA (Atomic and alternative energies commission), France

## Abstract

Pine wilt disease (PWD) is native to North America and has spread to Asia and Europe. Lately, mutualistic relationship has been suggested between the pinewood nematode (PWN), *Bursaphelenchus xylophilus* the causal nematode agent of PWD, and bacteria. In countries where PWN occurs, nematodes from diseased trees were reported to carry bacteria from several genera. However no data exists for the United States. The objective of this study was to evaluate the diversity of the bacterial community carried by *B. xylophilus*, isolated from different *Pinus* spp. with PWD in Nebraska, United States. The bacteria carried by PWN belonged to *Gammaproteobacteria* (79.9%), *Betaproteobacteria* (11.7%), *Bacilli* (5.0%), *Alphaproteobacteria* (1.7%) and *Flavobacteriia* (1.7%). Strains from the genera *Chryseobacterium* and *Pigmentiphaga* were found associated with the nematode for the first time. These results were compared to results from similar studies conducted from other countries of three continents in order to assess the diversity of bacteria with associated with PWN. The isolates from the United States, Portugal and China belonged to 25 different genera and only strains from the genus *Pseudomonas* were found in nematodes from all countries. The strains from China were closely related to *P. fluorescens* and the strains isolated from Portugal and USA were phylogenetically related to *P. mohnii* and *P. lutea*. Nematodes from the different countries are associated with bacteria of different species, not supporting a relationship between PWN with a particular bacterial species. Moreover, the diversity of the bacteria carried by the pinewood nematode seems to be related to the geographic area and the *Pinus* species. The roles these bacteria play within the pine trees or when associated with the nematodes, might be independent of the presence of the nematode in the tree and only related on the bacteria's relationship with the tree.

## Introduction

Pine Wilt Disease (PWD) is caused by the pinewood nematode (PWN), *Bursaphelenchus xylophilus*, first described in the USA as *Aphelenchoides xylophilus* and again, as *B. lignicolus*, when determined to be the causal agent of pine wilt disease in Japan and finally as *B. xylophilus* by Nickle and collaborators.[1]. Until now, is the only known causal agent of pine wilt disease. *B. xylophilus* is Native to North America (USA and Canada), it was introduced to Japan at the beginning of 20^th^ century and it has spread to China, Korea, Taiwan and recently to Europe (Portugal and Spain) [2]. PWN is causing one of the most devastating diseases in genus *Pinus* and has caused environmental and economic losses totaling multi-million US dollars around world [3]. The susceptible tree hosts to PWN are mainly conifers of the genus *Pinus*, with Pinus-species differing between geographical locations, such as *P. bunjeana*, *P. densiflora*, *P. luchuensis*, *P. massoniana* and *P. thunbergii* for Far Eastern countries and *P. nigra*, *P. sylvestris* and *P. pinaster* for European species [4]. In Portugal, *P. pinaster* is the only species known to be susceptible to PWN [5]. The transmission of *B. xylophilus* from tree to tree is accomplished by insect vectors of the genus *Monochamus* primarily, during the feeding. Different strategies have been used to address the challenge of PWD [6], [7]. The role of the endophytic bacterial community in pine trees have been studied [8]–[12] since the presence of endophytes [13] in plant tissues has been recognized as relevant for the trees [14], [15]. Some studies have indicated that bacteria may play a role in PWD [8], [16]–[19]. In different countries affected by PWD, different bacterial genera have been isolated associated with *B. xylophilus*. In Japan, strains from the genus *Bacillus* [20] and from the genus *Pseudomonas* [21] were identified to be associated with PWN. Moreover, researchers in China isolated strains from different genera which the most frequently isolated genus was *Pseudomonas* [16], [22]–[24]. In Republic of Korea, the bacterial genus associated to PWN found in common with China was *Serratia* [25]. Recently, it was shown that in Portugal bacteria associated with PWN were mainly belonging to the genera *Pseudomonas*, *Burkholderia*, and to the family *Enterobacteriaceae* [8], [26]. Others suggested that bacteria, carried by the PWN, are phytotoxin producers and interact with the nematode. If true, this may presumably result from a long-term co-evolution between the nematode and the bacteria [22], [27].

The methodologies used to identify the bacterial isolates recovered from the nematodes varied between the different studies. Some studies characterized their isolates by cultivation methods and identification kits [16], [20]–[25], [28] and other sequenced the 16S rRNA genes and queried them with the international databases [8], [12], [26], [28], [29]. Therefore, identifications of the isolated strains obtained only by biochemical methods may be incomplete, especially for some of the genera, potentially leading to wrong conclusions regarding bacterial communities.

The objective of this study was to evaluate, the diversity of the bacterial community carried by *B. xylophilus* in the United States from different tree species of the genus *Pinus* with PWD, and to identify a bacterial species that is commonly associated with all *B. xylophilus*, suggesting a privileged relationship with a role in PWD.

## Material and Methods

### Ethics Statement

No specific permissions were required for sampling and authorizations were obtained by Dr. Mark Harrell, Forest Health Specialist, UNL, USA. The field studies did not involve endangered or protected species.

### Sampling areas

Sampling was performed on June of 2011. *Pinus* trees from five different areas affected by PWD in Nebraska, USA were sampled: large golf course (G) (40°46′27.92″N, 96°38′39″W), small Golf course (GS) (40°47′30.81N, 96°40′35″W), Denis Land (D) (40°59′50.70″N, 96°44′14.36″W), Walter Land (W) (40°50′31.2″N, 96°33′54″W) and University of Nebraska-Lincoln East Campus (UNL) (40°49′54.03″N, 96°39′41′65″W) (Table 1). The sampling areas included pine trees with and without pine wilt disease, except the golf course where all the pine trees were healthy. G and Gs were golf courses with grass, and scattered pine trees from the species *P. sylvestris*. D and W were woodland areas, with different pine tree species. UNL was grass land with trees from different species, in groups. In area G, six symptomatic *P. sylvestris* trees and one asymptomatic *P. sylvestris* tree were sampled. In area GS, two symptomatic *P. sylvestris* trees were sampled. In area D, four symptomatic pine trees, three *P. sylvestris* and one *P. nigra*, and three asymptomatic pine trees, two *P. sylvestris* and one *P. nigra*, were sampled. In area W, one asymptomatic *P. ponderosa* tree and eight symptomatic pine trees, four *P. nigra* and four *P. sylvestris* were sampled. In the UNL area, four symptomatic pine trees, one *P. nigra* and three *P. sylvestris*, and one asymptomatic *P. ponderosa* tree were sampled (Table 1).

**Table 1:** Sampled *Pinus* spp. from different geographical areas and classified based on the PWD symptoms they expressed. +, presence of nematode; -, absence of nematode.

Sampling Area	Sample Tree	Pine tree species	PWD symptom class^a^	*B. xylophilus*	Other nematodes^b^
	Arv1	*P. sylvestris*	V	-	-
	Arv2	*P. sylvestris*	0	-	-
Golf course (G)	Arv3	*P. sylvestris*	I	-	-
	Arv4	*P. sylvestris*	V	-	-
	Arv6	*P. sylvestris*	II	-	-
	Arv7	*P. sylvestris*	III	-	-
Golf course small (Gs)	Arv8	*P. sylvestris*	V	-	-
	Arv9	*P. sylvestris*	III	-	-
	Arv10	*P. sylvestris*	V	+	-
	Arv11	*P. sylvestris*	III	+	-
	Arv12	*P. sylvestris*	III	-	-
Denis Land (D)	Arv13	*P. nigra*	V	+	A
	Arv14	*P. nigra*	0	-	-
	Arv15	*P. sylvestris*	0	-	-
	Arv16	*P. sylvestris*	0	-	-
	Arv17	*P. nigra*	IV	-	-
	Arv18	*P. sylvestris*	IV	-	-
	Arv19	*P. ponderosa*	0	-	-
	Arv20	*P. nigra*	V	+	-
Walter Land (W)	Arv26	*P. sylvestris*	V	-	-
	Arv27	*P. sylvestris*	IV	-	-
	Arv28	*P. sylvestris*	V	+	-
	Arv29	*P. nigra*	V	+	-
	Arv30	*P. nigra*	V	-	-
	Arv21	*P. ponderosa*	0	-	-
UNL East Campus	Arv22	*P. sylvestris*	V	+	R
(UNL)	Arv23	*P. sylvestris*	V	+	-
	Arv24	*P. sylvestris*	V	-	-
	Arv25	*P. nigra*	V	+	-

a 0 – tree without symptoms, I - <10% brown leaves, II - 10–50% brown leaves, III – 50–80% brown leaves, IV - >80% brown leaves, V- dead tree without leaves.

b A- *Aphelenchoidae* and R- *Rhabditidae*

### Plant material

Each sample consisted of pinewood cross-sections from cut trees or wood obtained by drilling a 5 mm diameter hole to a depth of 10 to 15 cm with a sterilized hand brace drill (Haglof, Mora, Sweden). The wood samples (1–3 cm^3^) were placed in labelled and sealed individual sterile plastic bags. All samples were kept at 4°C and analyzed within 24 h. The trees were classified into 6 symptom classes previously defined [8], based on the symptoms they expressed.

### Microbial community carried by PWN

The bark and sapwood of each sample were removed under aseptic conditions and the wood cut in ca. 2 cm chips. The wood pieces were placed onto R2A agar plates and incubated at 25°C, for three days. All bacterial colonies were isolated from the trails made by the nematodes on the medium [8]. The R2A agar (Reasoner's 2A agar, Difco) plates without nematodes were also used for isolation of bacteria. Bacterial isolates were grouped by RAPD typing. RAPD fragments were amplified by PCR, using primer OPA-03 (5′ – AGT CAG CCA C – 3′) (Operon Technologies, California, USA) together with crude cell lysates. DNA profiles for 107 isolates were grouped on basis of visual similarities of the fragments analyzed by electrophoresis in a 2% agarose gel stained with ethidium bromide. Reproducibility of the patterns was confirmed.

### Nematode screening and identification

Nematodes were removed from R2A medium plates and collected into 250 µL tubes with dH_2_O. The nematodes were observed using an inverted stereomicroscope, 200x magnification (Olympus CKX41SF, Tokyo, Japan). The identification of PWN and other nematodes was based on their diagnostic morphological characters. Identification of *B. xylophilus* was verified molecularly by a satellite-DNA species-specific based technique [30].

### Determination of 16S rRNA gene sequences of bacterial isolates from nematode trails

Amplification of a nearly full-length 16S rRNA gene sequence from bacterial isolates was performed by PCR with primers 27F (5′ – GAG TTT GAT CCT GGC TCA G – 3′) and 1525R (5′ – AGA AAG GAG GTG ATC CAG CC – 3′) [31]. The PCR reaction mix (50 µl) contained: reaction buffer (1.5 mM MgCl_2_, 50 mM KCl and 10 mM Tris-HCl, pH 8.3), 100 µM (each) deoxynucleoside triphosphates (NZYTech, Lisbon, Portugal), 0.2 µM (each) primer and 1.5 U Supreme NZYTaq DNA polymerase (NZYTech). The PCR was performed with 30 cycles: 1 min at 94°C, 1 min at 53°C, and 1 min at 72°C.PCR products with 1,500 bp obtained from isolates were purified using the NZYGelpure kit (NZYTech) according to the manufacturer's instructions, and sequenced as described below.

### DNA sequence analysis and phylogenetic analysis

The 16S rRNA genes, from RAPD-types representing all different strains, were subjected to amplification for sequencing. Automated sequencing of the purified PCR products was performed by Eurofins MWG Operon (California, USA).

All sequences were compared with sequences available in the EMBL/GenBank database using BLASTN network services [32] and with sequences in the Eztaxon-e server (http://eztaxon-e.ezbiocloud.net/) [33]. Sequences were aligned within the SINA alignment service [34] and checked for chimeric artefacts by using Mallard software [35]. Phylogenetic dendrograms were constructed by the maximum likelihood (RAxML) method included inside ARB software [36] and also the neighbor-joining phylogenetic trees were constructed by using Jukes-Cantor method [37] included in MEGA 5 software [38]. Bootstrap analysis with 1,000 replicates was used to evaluate the robustness of the phylogeny.

All sequences from this study were compared with sequences from strains carried by wild *B. xylophilus* (isolated from trees), present in the EMBL/GenBank database and included in previous publications. A total of 98 sequences were found: 60 from Portugal in 2010 [8]; 21 from Portugal in 2011 [26]; 2 from China in 2012 [29] and 15 from China in 2013 [12]. The sequences were aligned and the dendrograms were constructed as mentioned above.

### Data analysis

Relationships between pine trees, bacterial species and the environmental variables (pine trees species including *P. nigra*, *P. sylvestris* and *P. ponderosa*; five sampling areas and PWD symptom class (Table 1) and *B. xylophilus* presence) were analyzed by redundancy analysis (RDA) using the software package CANOCO (version 4.5.1).

RDA was accompanied by Monte Carlo permutation tests to evaluate the statistical significance of the effects of the explanatory variables on the species composition of the samples [39].

### Nucleotide sequence accession numbers

The 16S rRNA gene sequences of the bacterial isolates carried by PWN reported in this study have been deposited in Genbank/EMBL database under the accession numbers KF214941-KF214978.

## Results

### Nematode screening and identification

The pinewood nematode *B. xylophilus* was detected in nine symptomatic pine trees (class III and V of PWD symptom class) from three of the five sampling areas (Table 1). *B. xylophilus* and nematodes from other families (Families *Aphelenchoididae* and *Rhabditidae*) were found in two symptomatic pine trees (Arv13 and Arv22) from the species *P. nigra* and *P. sylvestris*, respectively. No nematodes were detected in the six asymptomatic trees.

### Microbial community carried by PWN

**Figure 1:**
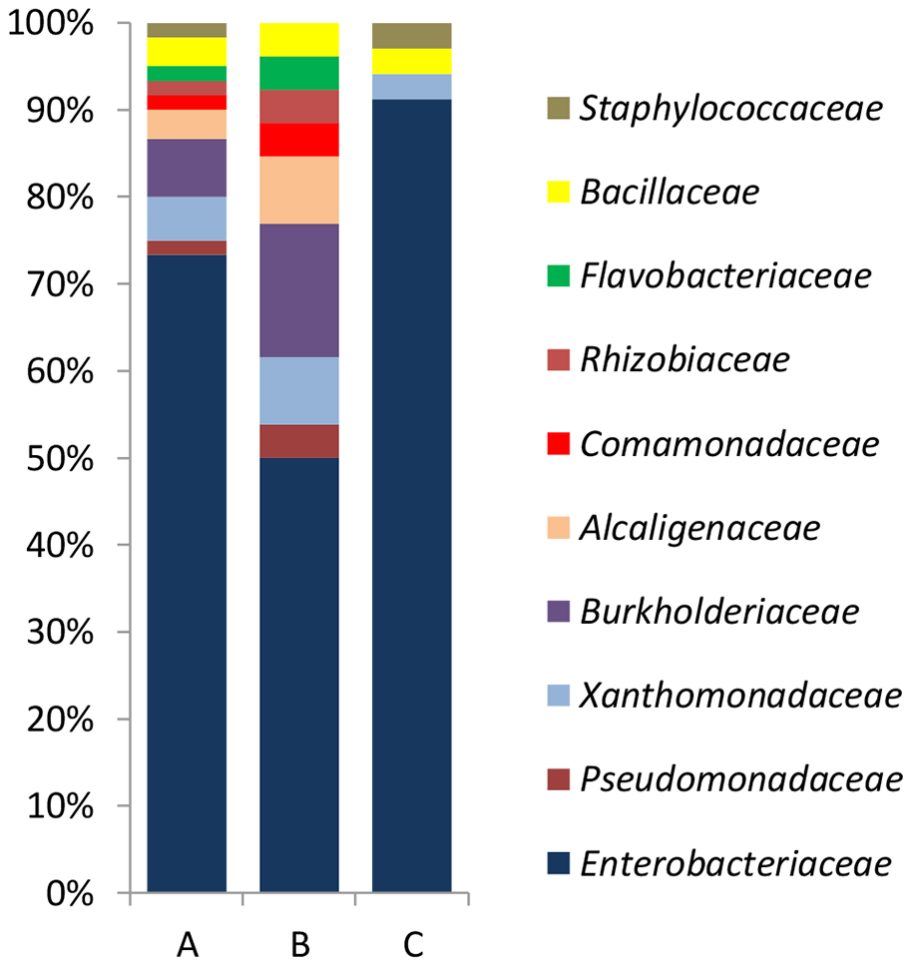
Microbial community composition, diversity and relative abundance of: total of bacteria carried by PWN (A), bacteria carried by PWN from *P. sylvestris* (B) and from *P. nigra* (C). The families: *Alcaligenaceae*, *Bacillaceae*, *Burkholderiaceae*, *Comamonadaceae*, *Enterobacteriaceae*, *Flavobacteriaceae*, *Pseudomonadaceae*, *Rhizobiaceae*, *Staphylococcaceae* and *Xanthomonadaceae*.

The microbial community carried by nematodes was isolated from nine pine trees (Table 1). A total of 107 strains were isolated of which forty-seven isolates were identified by microscopy as yeasts. Bacterial strains carried by PWNs were found in four *P. sylvestris* trees (Arv-11, -22, -23, and -28) and in four *P. nigra* (Arv-13, -20, -25, and -29) and all the strains were isolated. Bacteria were not isolated from the other nematode species. The bacterial strains were grouped into 38 RAPD-types on basis of visual similarities, representing a total of 60 bacterial strains. Based on identification using international databases, these bacterial strains belonged to six phylogenetic classes: *Alphaproteobacteria* (1.7%), *Betaproteobacteria* (11.7%), *Gammaproteobacteria* (79.9%), *Flavobacteriia* (1.7%) and *Bacilli* (5.0%) (Figure 1). A detailed description of the bacterial families and genera carried by PWN, from the different pine species, and their frequencies, is presented in Table 2.

**Table 2:** Frequencies of bacterial taxa carried by *B. xylophilus* isolated from different *Pinus* spp.

Class	Frequencies (%)	Family	Frequencies (%)	Genera	Frequencies (%) from *P. sylvestris*	Frequencies (%) from *P. nigra*
*Alphaproteobacteria*	1.7	*Rhizobiaceae*	1.7	*Rhizobium*	1.7	0
*Bacilli*	5.0	*Bacillaceae*	3.3	*Bacillus*	1.7	1.6
		*Staphylococcaceae*	1.7	*Staphylococcus*	0	1.7
		*Burkholderiaceae*	6.7	*Burkholderia*	6.7	0
*Betaproteobacteria*	11.7	*Alcaligenaceae*	3.3	*Pigmentiphaga*	3.3	0
		*Comamonadaceae*	1.7	*Comamonas*	1.7	0
*Flavobacteriia*	1.7	*Flavobacteriaceae*	1.7	*Chryseobacterium*	1.7	0
				*Serratia*	5.0	8.3
				*Ewingella*	0	18.3
		*Enterobacteriaceae*	73.3	*Enterobacter*	1.7	0
				*Mangrovibacter*	0	1.7
*Gammaproteobacteria*	79.9			*Klebsiella*	15.0	3.3
				*Erwinia*	0	20.0
		*Pseudomonadaceae*	1.6	*Pseudomonas*	1.6	0
		*Xanthomonadaceae*	5.0	*Dyella*	1.7	0
				*Pseudoxanthomonas*	1.7	1.6

**Figure 2:**
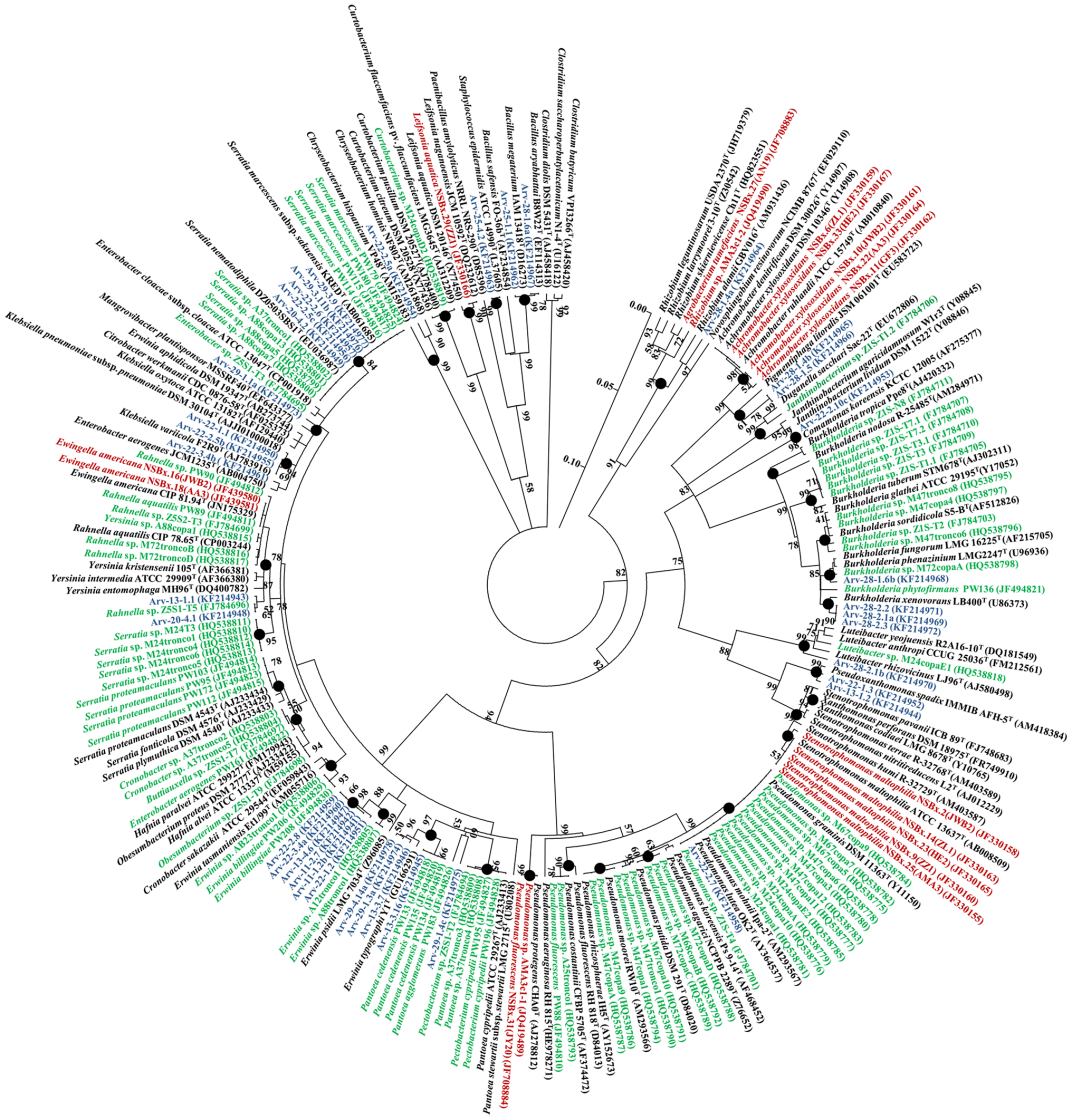
Phylogenetic analysis of bacterial 16S rRNA gene sequences of bacteria carried by PWN obtained from different countries (China - red, Portugal - green; USA - blue) and sequences available from NCBI. The circular tree was generated using a neighbor-joining analysis included in MEGA 5 software, partial deletion (95%), linearized and rooted by *Clostridium* spp. Symbol (•) indicates node branches conserved when the tree was reconstructed using the maximum likelihood (RAxML). The numbers on the tree indicate the percentages of bootstrap sampling, derived from 1,000 replications, values below 50% are not shown. Isolates characterized in this study are indicated in blue. Scale bar, 5 inferred nucleotide substitutions per 100 nucleotides.

Some of the strains were identified to the species level. The most abundant species in the family *Enterobacteriaceae* were *Ewingella americana* and *Erwinia typographi* representing 50% of the diversity inside the family, followed by the species *S. marcescens* (21%) that was the second most abundant (Figure 2, Figure S1). The class *Betaproteobacteria* was represented by strains of *Burkholderia xenovorans* (43%) and *B. phenazinium* (14%), two strains of the species *Pigmentiphaga litoralis* and one identified as *Comamonas koreensis*. *Flavobacteriia* was represented by one strain of species *Chryseobacterium hominis*. Bacteria inside *Firmicutes* were identified as *Bacillus megaterium* (66.7%) and *Staphylococcus epidermidis* (33.3%).

Comparing all the sampling areas, the microbial community carried by the nematodes was different for each sampling area. In the G area, only yeasts but no bacteria were detected associated with nematodes. The genus *Bacillus* was found in the W and UNL sampling areas but just in nematodes from the pine *P. sylvestris*. The species *Pseudoxanthomonas spadix* was common to D and UNL sampling areas. Strains from the family *Enterobacteriaceae* were present in UNL, D and A areas. Although, strains from the genera *Pseudomonas* and *Burkholderia* were only found in UNL and W sampling areas, respectively.

### Relationships between pine trees species, bacterial communities and environmental variables

**Figure 3:**
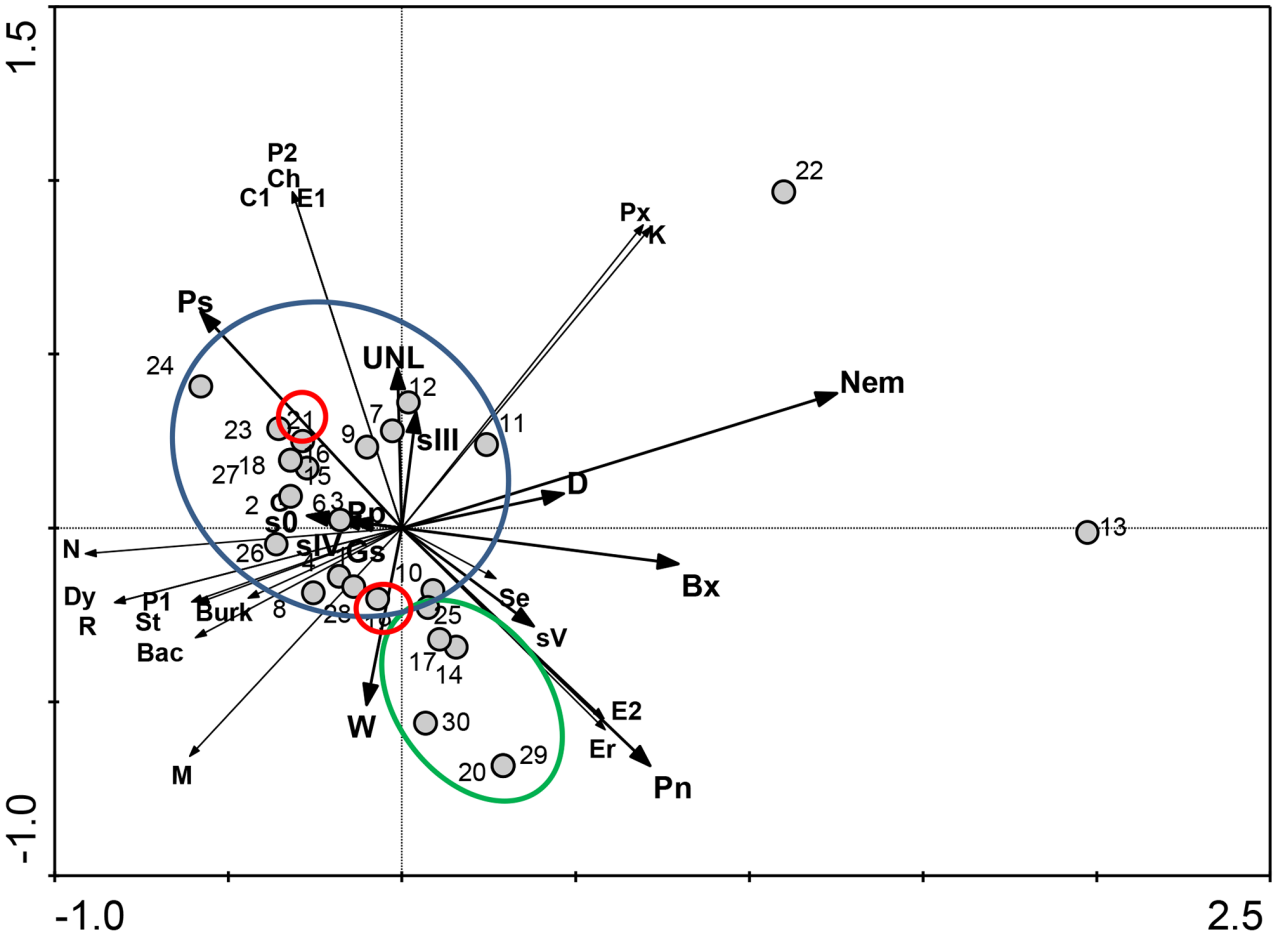
Relationships between pine trees species, bacterial communities and environmental variables. Redundancy analysis (RDA) performed with bacterial communities carried by PWN from five sampling sites. Circles highlight the closest relationship between *P. sylvestris* (blue), *P. nigra* (green) and *P. ponderosa* (red). The cumulative percentage of first and second axis explained 80.4% of variance. The genera included in this analysis are: Bac - *Bacillus*, Burk - *Burkholderia*, C1 - *Comamonas*, Ch - *Chryseobacterium*, Dy - *Dyella*, E1 - *Enterobacter*, E2 - *Ewingella*, Er - *Erwinia*, K - *Klebsiella*, M - *Mangrovibacter*, P1 - *Pigmentiphaga*, P2 - *Pseudomonas*, Px - *Pseudoxanthomonas*, R - *Rhizobium*, Se - *Serratia*, St - *Staphylococcus*. The environmental variables are: sampling areas (G, Gs, W, D, UNL), pine tree species (Pn – P. nigra, Pp – *P. ponderosa*, Ps – *P. sylvestris*), presence of *B. xylophilus* (Bx) or other nematodes (Nem), and PWD symptom classes (s0, sIII, sIV, sV). The numbers are the pine trees sampled (Table 1).

In order to understand the relationships between pine tree species and bacterial communities carried by nematodes in different environmental conditions, the data obtained were explored by RDA (Figure 3). The first and second axes explained 53.4% and 80.4% of cumulative variation, respectively. The first axis, representing the direction of maximum variation through the data, seems to separate the pine trees, based on the presence or absence of nematodes. The analysis revealed two main groups, one constituted by *P. sylvestris* trees and the other by *P. nigra* trees (Figure 3). However, the two pine trees of species *P. ponderosa* are included in group of *P. sylvestris* trees. Moreover, the pine trees 13 (*P. nigra*) and 22 (*P. sylvestris*) are outsiders, most probably due to the presence of other nematodes but they are separated depending on their *Pinus* species.

### Phylogenetic relationship between strains carried by *B. xylophilus* from different countries

All the 16S rRNA sequences from bacterial strains carried by *B. xylophilus* from all the different countries with available data (USA, Portugal and China), with accession numbers mentioned in the literature and found in the NCBI database, were considered in a total of 136 sequences. Strains from Portugal were isolated using the same methodology as the one used in this study [8], or by using several selective (*Pseudomonas* complex medium) and non-selective media (Trypticase Soy Agar (TSA), Nutrient Agar (NA), Luria Agar (LA)) incubated at 28°C for 1 week [26]. The strains from China were isolated using NA or Nutrient Broth (NB) incubated for 2–3 days at 30°C [12], [29]. The majority of the strains belonged to the class *Gammaproteobacteria*, others belonged to the class *Betaproteobacteria* and some strains to the class *Alphaproteobacteria*. Thirteen strains isolated from USA did not group with other isolates and belonged to the species *Mangrovibacter plantisponsor* (EF643377) (Arv-29-1.1a), *Klebsiella variicola* (AJ783916) (Arv-22-1.1, Arv-22-2.5b), *Erwinia typographi* (GU166291) (Arv-29-1.3a, Arv-29-4.14a, Arv-13-3.7), *Pigmentiphaga litoralis* (EU583723) (Arv-28-1.4b, Arv-28-1.5), *Comamonas koreensis* (AF275377) (Arv-22-2.10c), *Pseudoxanthomonas spadix* (AM418384) (Arv-13-1.2, Arv-22-1.3), *Chryseobacterium hominis* (AM261868) (Arv-22-2.5a), and *Bacillus megaterium* (D16273) (Arv-25-1.1, Arv-28-1.6a). The clade grouping strains from the genera *Ewingella* and *Rahnella* included strains from the 3 countries analyzed. Bacteria from the genera *Serratia* and *Pantoea* were both grouped into 2 subgroups and did not included strains from China. The genus *Pseudomonas* grouped strains from the 3 countries. Strains from the genus *Burkholderia* grouped strains from Portugal and USA. Most isolates from China belonged to the genus *Achromobacter* and grouped together with 2 strains of *Pigmentiphaga* from USA, both belonging to the *Alcaligenaceae*. Another 2 strains from USA belonged to *Pseudoxanthomonas* and grouped with the *Stenotrophomonas* strains from China.

## Discussion

It is assumed that in Pine Wilt Disease, *B. xylophilus* is the causal agent but bacteria, according to recent findings [22], may play a role in the disease progression, as bacteria from several genera have been found associated with the PWN.

Functional relationships have been suggested between *B. xylophilus* and bacteria [40]. Further, a genetic relationship between the two groups is supported by the presence of prokaryotic genes in the genome of nematodes from different families suggesting a co-evolutionary process with horizontal gene transfer [41]–[43]. Until now, different studies have demonstrated that PWN, obtained from infected trees from different countries, have an associated bacterial community [8], [12], [16], [20], [23], [25], [26], [29]. However, the comparison between the bacterial species associated with the nematodes from different countries is very difficult because the bacterial identification of most of the works were based on phenotypic characterization, using different methodologies, and not based on the 16S rRNA gene sequence of the strains. In the present work, all the bacterial strains carried by *B. xylophilus*, with public accession numbers in the NCBI database, were included for comparison of the bacterial communities. Only strains from USA, China and Portugal were found in the database and compared phylogenetically. Although, different methodologies were used for bacterial isolation all were based on non-selective media at similar incubation temperature that potentiating the isolation of mesophilic bacteria [8], [12], [26], [29].

In the USA, as in Portugal, most of the bacterial strains carried by PWN belonged to the family *Enterobacteriaceae* independent of the sampling area, and *Burkholderiaceae* were only found associated with nematodes from one area [8]. Moreover, strains from the genera *Chryseobacterium* and *Pigmentiphaga* were found associated with the nematodes, for the first time in USA. Nevertheless, when considering the different parameters, the PWN-associated bacterial populations seemed to be related with the geographical area as well as pine tree species.

Most of the bacterial genera carried by USA nematodes are strains described as plant associated organisms, either plant growth promoting or plant pathogens, some genera including species with opposite functions. Strains of *Achromobacter xylosoxidans* (family *Alcaligenaceae*) and from *Stenotrophomonas maltophilia* (family *Xanthomonadaceae*) are versatile, able to degrade aromatic molecules such as pyrene. Both species were isolated from *B. xylophilus* strains considered having moderate virulence [12], [44]. *Bacillus megaterium* also isolated in association with the nematodes, is a phosphate solubilizing bacterium, considered plant growth promoting [45]. On the other hand, most of the strains from the genera *Pantoea* and *Erwinia* are phytopathogens. So, if PWNs are carrying bacteria from tree to tree (inside the insect vector) they could at the same time, introduce beneficial and harmful bacteria into the pine trees. However, recently studies showed that some strains from the genera *Serratia* and *Pseudomonas* have a nematicidal activity against *B. xylophilus* [9], [46], [47]. Thus, a third role can be envisaged for the associated bacteria which is their negative activity against PWN.

The bacterial strains associated with PWN found in the NCBI database were from China, Portugal and USA, and belonged to 25 different genera. However, only strains from the genus *Pseudomonas* were isolated from nematodes of each country. *Pseudomonas* is a very diverse group including species with very different functional characteristics. The strains from China were closely related to *P. fluorescens* and did not group with *Pseudomonas* strains isolated from Portugal or USA, which in turn were most related with the species *P. mohnii* and *P. lutea*. The *P. fluorescens* strain AMA31c1-1 [29] grouped with the *P. fluorescens* strain NSBx.31 [12] from China. Han and coworkers (2003) suggested that *P. fluorescens* may be correlated with nematode virulence [16], but strain NSBx.31 [12] had no visible effects on the virulence of PWN and strain AMA3c1-1 protected the plant against PWD [29].

Taking in consideration all variables, the pine trees were statistically grouped in two major groups which seem to be related with tree species (although, *P. ponderosa* grouped with *P. sylvestris*), the presence of some bacterial genera and the presence of other nematodes.

In conclusion, nematodes from the different countries are associated with bacteria of different species, not supporting a relationship between the nematode with a particular bacterial species. In addition, the diversity of the bacteria carried by the pinewood nematode seems to be dependent on the geographic area and the pine tree host species. The role of the different bacteria species inside the tree is probably independent of the presence of the nematode and only dependent on the bacteria's relationship with the tree.

## Supporting Information

Figure S1 **Phylogenetic analysis of bacterial 16 S rRNA gene sequences of bacteria carried by PWN obtained from different countries (China - red, Portugal - green; USA - blue) and sequences available from NCBI.** The dendrogram was constructed by the RAxML method with GTRGAMMA model included inside ARB software and rooted by *Clostridium* spp. Symbol (•) indicates node branches conserved when the tree was reconstructed using the neighbor-joining method. The numbers on the tree indicate the percentages of bootstrap sampling, derived from 1,000 replications, values below 50% are not shown. Isolates characterized in this study are indicated in blue. Scale bar, 1 inferred nucleotide substitution per 100 nucleotides.
(PDF)
