# Supplementary figures and images for: Diversity of Bacteria Carried by Pinewood Nematode in USA and Phylogenetic Comparison with Isolates from Other Countries
Source: PLoS One. 2014 Aug 15;9(8):e105190. doi: 10.1371/journal.pone.0105190 (PMC4134288; doi:10.1371/journal.pone.0105190)

Figure S1.

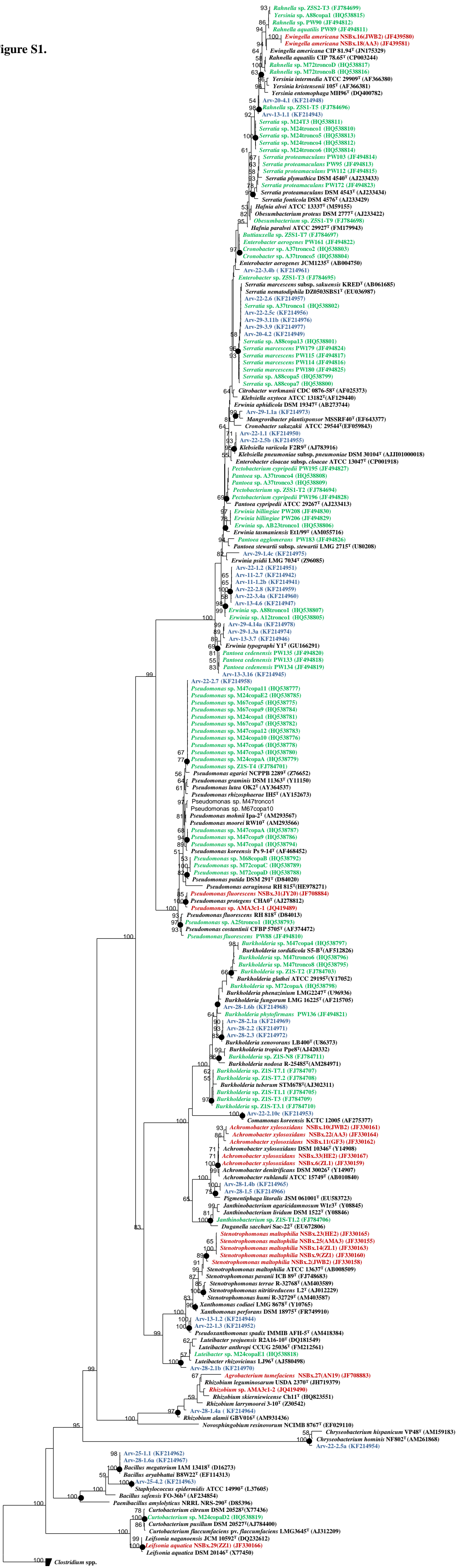

Supplement: Figure S1 — Phylogenetic analysis of bacterial 16 S rRNA gene sequences of bacteria carried by PWN obtained from different countries (China - red, Portugal - green; USA - blue) and sequences available from NCBI. The dendrogram was constructed by the RAxML method with GTRGAMMA model included inside ARB software and rooted by Clostridium spp. Symbol (•) indicates node branches conserved when the tree was reconstructed using the neighbor-joining method. The numbers on the tree indicate the percentages of bootstrap sampling, derived from 1,000 replications, values below 50% are not shown. Isolates characterized in this study are indicated in blue. Scale bar, 1 inferred nucleotide substitution per 100 nucleotides. (PDF) [file pone.0105190.s001.pdf]
